# Relationship between the Amount of Bitter Substances Adsorbed onto Lipid/Polymer Membrane and the Electric Response of Taste Sensors

**DOI:** 10.3390/s140916274

**Published:** 2014-09-02

**Authors:** Kiyoshi Toko, Daichi Hara, Yusuke Tahara, Masato Yasuura, Hidekazu Ikezaki

**Affiliations:** 1 Graduate School of Information Science and Electrical Engineering, Kyushu University, Nishi-ku, Fukuoka 819-0395, Japan; E-Mails: toko@ed.kyushu-u.ac.jp (K.T.); hara@nbelab.ed.kyushu-u.ac.jp (D.H.); yasuura@belab.ed.kyushu-u.ac.jp (M.Y.); 2 Research and Development Center for Taste and Odor Sensing, Kyushu University, Nishi-ku, Fukuoka 819-0395, Japan; 3 Intelligent Sensor Technology, Inc., 5-1-1 Onna, Atsugi-shi, Kanagawa 243-0032, Japan; E-Mail: Ikezaki.Hidekazu@insent.co.jp

**Keywords:** taste sensor, electronic tongue, global selectivity, CPA measurement, adsorption amount

## Abstract

The bitterness of bitter substances can be measured by the change in the membrane electric potential caused by adsorption (CPA) using a taste sensor (electronic tongue). In this study, we examined the relationship between the CPA value due to an acidic bitter substance and the amount of the bitter substance adsorbed onto lipid/polymer membranes, which contain different lipid contents, used in the taste sensor. We used *iso*-α-acid which is an acidic bitter substance found in several foods and beverages. The amount of adsorbed *iso*-α-acid, which was determined by spectroscopy, showed a maximum at the lipid concentration 0.1 wt % of the membrane, and the same phenomenon was observed for the CPA value. At the higher lipid concentration, however, the amount adsorbed decreased and then remained constant, while the CPA value decreased monotonically to zero. This constant adsorption amount was observed when the membrane potential in the reference solution did not change with increasing lipid concentration. The decrease in CPA value in spite of the constant adsorption amount is caused by a decrease in the sensitivity of the membrane as the surface charge density increases. The reason why the peaks appeared in both the CPA value and adsorption amount is based on the contradictory adsorption properties of *iso*-α-acid. The increasing charged lipid concentration of the membrane causes an increasing electrostatic attractive interaction between iso-α-acid and the membrane, but simultaneously causes a decreasing hydrophobic interaction that results in decreasing adsorption of *iso*-α-acid, which also has hydrophobic properties, onto the membrane. Estimates of the amount of adsorption suggest that *iso*-α-acid molecules are adsorbed onto both the surface and interior of the membrane.

## 1. Introduction

Taste as identified by humans consists of five basic taste qualities: saltiness, sourness, umami, sweetness, and bitterness. Among these tastes, bitterness is a signal of toxicity for living organisms, and hence is an unpleasant taste for many people, affecting the value of food products. Therefore, evaluating bitterness is an important task in food and pharmaceutical industries, and is useful for developing new products. Sensory tests, in which panelists actually taste samples to evaluate them, have some problems such as low objectivity and reproducibility, as well as the stress possibly imposed on panelists. In the pharmaceutical industry, it is not easy to perform sensory tests because of the possibility of side effects. Quantitative analysis carried out on bitter substances contained in drugs cannot reveal the strength of bitterness. With this background, sensors that can objectively evaluate taste are desirable and have been developed [1–14].

Electronic tongues and a taste sensor, which is a kind of electronic tongue, have been used in the research and development of new products and their quality management in the food and pharmaceutical industries [1–10,15–19]. The taste sensor utilizes lipid/polymer membranes as the taste-sensing part and outputs a change in the membrane potential caused by the interaction between the lipid/polymer membrane and taste substances. Lipid/polymer membranes are composed of a polymer support, a lipid, and a plasticizer. The lipid and plasticizer are responsible for controlling hydrophobic and electrical properties of the membrane surface, whereas the polymer support plays the role in encapsulating and immobilizing the lipid. Lipid/polymer membranes are designed and developed so that they can selectively respond to each taste quality. This characteristic that the sensor can decompose chemical substances into each type of taste by mimicking the human tongue is called global selectivity [1,2,4–6,9–13].

The lipid/polymer membrane responds to taste substances in sample solutions, and this response is called relative value, which is the difference between the membrane potential for the sample solution and that for the reference solution as the origin. Bitter substances, most of which are hydrophobic substances, are adsorbed onto the lipid/polymer membrane to cause a change in the membrane electric potential. The change in the membrane potential caused by adsorption (CPA) is used as an index of the aftertaste of samples felt by humans [1,2,4,6,10,12,20]. Only adsorptive taste substances can be targeted in the CPA measurement, which enables the lipid/polymer membrane to respond selectively to bitter substances.

Relationships between the CPA value and adsorbed amount of taste substances have been studied for basic bitterness (quinine) and astringency (as the sixth taste quality: tannic acid) [21,22]. As a result, it was confirmed that the CPA value is directly affected by the amount of taste substances adsorbed onto the membrane, as suggested in the past [20]. The peaks of both the CPA value and the adsorbed amount appeared around the same lipid concentration as high as 10 wt % of the membrane. In the present paper, the similar method is applied to acidic bitterness (*iso*-α-acid).

A short report on *iso*-α-acid using a membrane composed of tetradodecylammonium bromide (TDAB) and 2-nitrophenyl octyl phosphate (NPOE) [2], which is used in the commercialized acidic bitterness sensor C00 (Intelligent Sensor Technology, Inc., Kanagawa, Japan) was recently published. This bitterness sensor is shown to respond to acidic bitter substances contained in beer, green tea, black tea, Oolong tea, coffee and wine [1,2,4]. The detection limit for *iso*-α-acid is about 0.001 wt %, which is close to the human threshold. A first report on the membrane composed of trioctylmethylammonium chloride (TOMA) and dioctylphosphonate (DOPP), which has similar properties to the commercialized acidic bitterness sensor C00, for investigation of response magnitude (*i.e.*, magnitude of relative value) was published 15 years ago [23,24]. Although neither the CPA value nor the adsorption amount was measured in this report, it was pointed out that the relative value is largest in the lipid-concentration region around 0.1 wt %, which is much different from the above result of 10 wt % for quinine and tannic acid. A new phenomenon can be expected at the lipid concentrations higher than 0.1 wt %, at which the largest response appeared. Previous studies [2,21,22] showed that the amount of adsorption increases together with the CPA value by increasing chemical substances such as quinine, tannic acid and *iso*-α-acid. However, what the CPA value reflects or how it appears has not been clarified. The study, therefore, using the lipid/polymer membrane composed of TOMA and DOPP might lead to comprehensive understanding of relationship among the CPA value, the relative value and the adsorption of taste substances from the electrochemical point of view.

The present study aimed at clarification of electrical response mechanism of taste sensor for adsorptive chemical substances. The increase in adsorption amount has been already known to occur with increasing *iso*-α-acid concentration [2,21,22]. Therefore, the used *iso*-α-acid concentration was fixed at 0.01 vol % in both the experiment using taste sensor and the measurement of adsorption amount. This concentration corresponds to ten times the human threshold, and hence humans can sense this bitter taste substantially. As a result, it was found that the amount of adsorbed iso-α acid decreased and then kept constant while the CPA value monotonically decreased to zero at the higher lipid concentration of the membrane. The CPA response of lipid/polymer membrane for adsorptive bitter substances was discussed from the viewpoints of both the sensitivity and the adsorption amount, which is related to the hydrophobicity and the charge density of the membrane.

## 2. Experimental Section

### 2.1. Reagents

Trioctylmethylammonium chloride (TOMA) was purchased from Tokyo Chemical Industry Co., Ltd. (Tokyo, Japan). Dioctyl phenylphoshonate (DOPP) was purchased from Sigma-Aldrich, Inc. (St. Louis, MO, USA). Polyvinyl chloride (PVC) was purchased from Wako Pure Chemical Industries, Ltd. (Osaka, Japan). Potassium chloride (KCl), sodium hydrogen L-glutamate monohydrate, sucrose and tartaric acid were purchased from Kanto Chemical Co., Inc. (Tokyo, Japan). *iso*-α-Acid was purchased from Intelligent Sensor Technology Inc. (Kanagawa, Japan). All aqueous solutions were prepared with distilled water.

### 2.2. Lipid/Polymer Membranes

The lipid/polymer membranes used in this study comprise TOMA as the lipid, DOPP (0.5 mL) as the plasticizer, and polyvinyl chloride (PVC, 400 mg) as the polymer support. The chemical species and concentrations of lipid and plasticizer are changed and controlled in different kinds of membranes to detect each taste quality, as detailed [1,2,4,6]. The membranes containing TOMA in large quantities are positively charged because of the positive charge of TOMA, which results in a positive membrane potential. Here we prepared the membranes with different lipid concentrations because the properties of the membranes such as the surface or interior charge density and hydrophobicity depend on the lipid concentration of the membrane, which affects the CPA value and the amount of adsorbed bitter substances. The lipid concentration is shown by wt% in comparison with the PVC content in a way similar to previous works [2,21,22], because the plasticizer concentration also differs in each membrane for each taste quality in spite of the constant PVC content, 400 mg. In the preset study, 0.01, 0.1, 1 and 10 wt % correspond to 0.005, 0.045, 0.451 and 4.334 wt % in comparison with the total composition, respectively. The procedures for fabricating the membranes follow those used in the previous studies [1,4,6].

### 2.3. Measurement Using Taste Sensor

The relative value and CPA value were measured using a taste sensing system (SA402B, Intelligent Sensor Technology, Inc.) composed of several sensor electrodes and a common reference electrode. Each sensor electrode, to which a lipid/polymer membrane is attached, measures changes in the membrane potential that is generated when the electrode is immersed in a sample. Each of four membranes taken from one Petri dish was pasted on each of four electrodes. The samples used in the measurement are summarized in Table 1.

**Figure 1.**
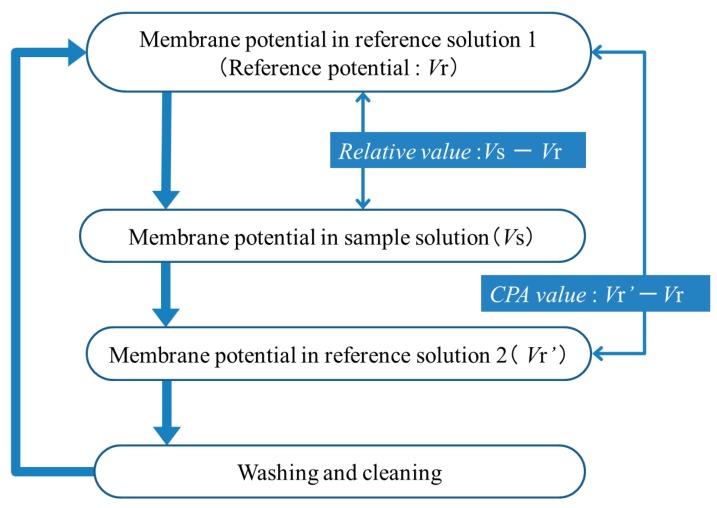
Measurement procedure using taste sensor.

The measurement procedure is shown in Figure 1. First, the sensor electrode is immersed in the reference solution 1 (30 mM KCl, 0.3 mM tartaric acid), and the membrane potential for the reference solution (reference potential), *V*r, is measured. Next, the sensor electrode is immersed in the sample solution, and the membrane potential (*V*s) is measured; the difference, *V*s − *V*r, is defined as the relative value. Then, the sensor electrode is again immersed in the reference solution 2 (30 mM KCl, 0.3 mM tartaric acid), and the membrane potential for the reference solution is measured again (*V*r′), and the difference between *V*r′ and *V*r, *i.e., V*r′ − *V*r, is defined as the CPA value. As detailed later, *V*r′ is different from *V*r, because iso-α acid keeps being adsorbed onto the membrane after measuring the sample solution when measuring the reference solution 2. Finally, the membrane is rinsed with a sensor rinsing solution (100 mM KCl, 10 mM KOH, 30 vol % ethanol). This procedure is repeated five times for each sample, and the averages of the relative values and CPA values in the second measurement to the fifth measurement using four membranes are used as the relative value and CPA value of each sample, respectively. The standard deviations were calculated for *n* = 4 (membranes) × 4 (rotations) = 16. The measurement time is 30 s in the reference solutions 1 and 2, and also the sample solution.

### 2.4. Measurement of Amount of Adsorbed iso-α-Acid

The amount of *iso*-α-acid adsorbed onto the lipid/polymer membrane was measured using an ultraviolet-visible spectrophotometer (UV-1800, Shimadzu Corporation, Kyoto, Japan) as described in previous reports [21,22]. First, the absorbance of an *iso*-α-acid solution of known concentration was measured to obtain a calibration curve showing the relationship between the concentration and absorbance. Five mL of *iso*-α-acid solution of a known concentration (bitterness solution in Table 1) was added dropwise onto a Petri dish on which a lipid/polymer membrane had been formed, and allowed to stand for 30 s to allow the *iso*-α-acid molecules in the solution to be adsorbed onto the membrane. After 30 s, 3 mL of the *iso*-α-acid solution was taken from the Petri dish to measure the absorbance of the solution. The concentration of *iso*-α-acid in the measured solution was calculated from the measured absorbance and calibration curve. The difference between the concentration of the *iso*-α-acid solution added dropwise and that of the solution after 30 s was defined as the amount of adsorbed *iso*-α-acid. This value was divided by the area of the Petri dish to obtain the amount of *iso*-α- acid adsorbed per square centimeter.

## 3. Results

### 3.1. Relative Values

**Figure 2.**
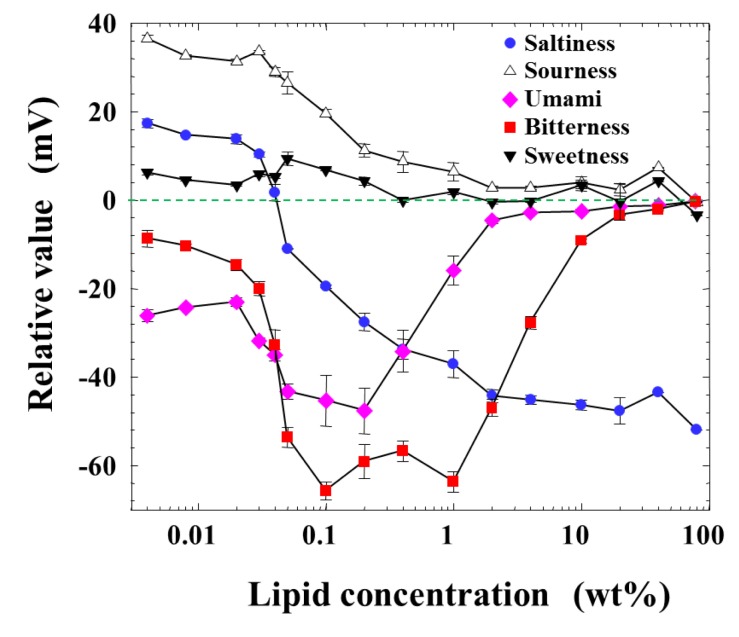
Responses (relative values) of the membrane to the basic taste substances as a function of the lipid concentration.

Figure 2 shows the measurement result of relative values for five basic taste substances as a function of the lipid concentration of the membrane from 0.004 to 80 wt %, which is the maximum amount to be dissolved in a solvent or formed as the membrane. The response to sweetness was nearly zero at every lipid concentration. The response to sourness decreased monotonically from about 40 mV to zero with the lipid concentration. While the relative value for saltiness was positive in the low-lipid-concentration region, it became zero around 0.04 wt % lipid concentration, and then turned to negative values with increasing lipid concentration. The peaks of relative values appeared broadly to the negative direction for bitterness and umami at the lipid concentration from 0.03 to 2 or 3 wt %.

### 3.2. CPA Values

**Figure 3.**
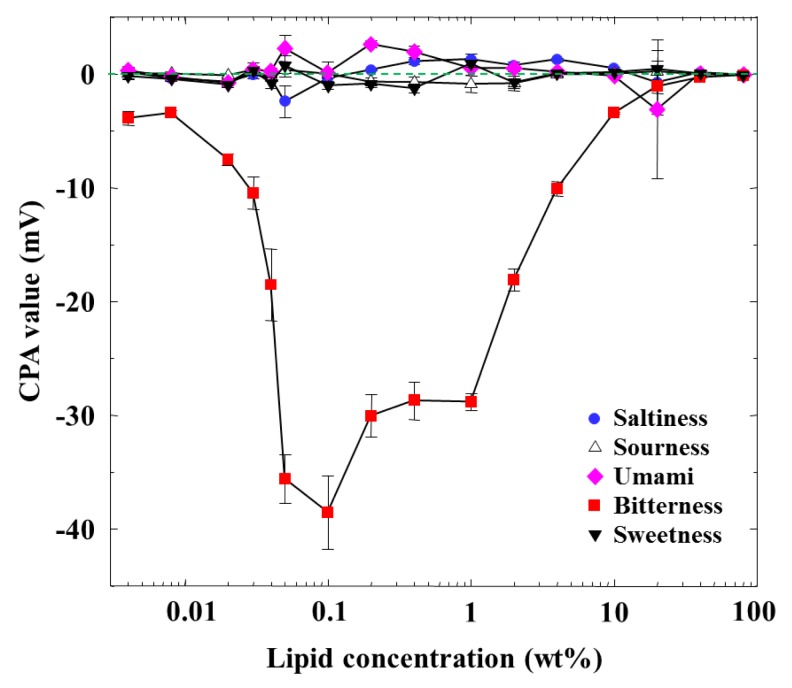
Responses (CPA values) of the membrane to the basic taste substances as a function of the lipid concentration.

Figure 3 shows the dependences of the CPA values of five basic taste substances on the lipid concentration. The CPA values of saltiness, sourness, sweetness, and umami were almost zero, even if the lipid concentration was changed. A CPA value only appeared for *iso*-α-acid (bitterness), implying a selective response to bitterness. The CPA value increased in the negative direction with the lipid concentration and was largest at 0.1 wt % concentration, and then its magnitude decreased gradually and finally became zero when the lipid concentration was further increased to 80 wt %. The CPA and relative values for bitterness behaved in a similar way, whereas they showed no similar tendency for the other taste qualities; this is a noticeable property of CPA measurement, as also explained [1,2,4,6,10,12,19].

### 3.3. Amount of Adsorbed iso-α-Acid

**Figure 4.**
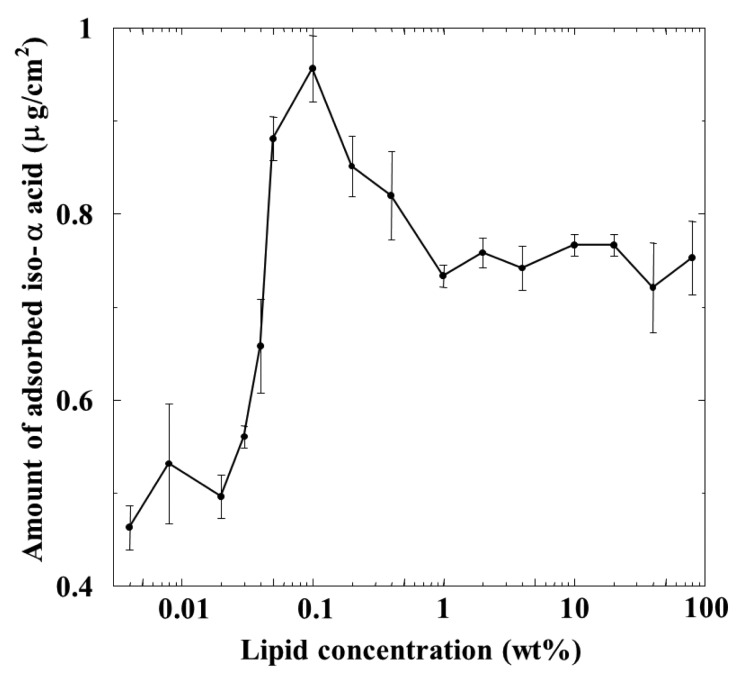
Relationship between the amount of adsorbed *iso*-α-acid and the lipid concentration.

The amount of *iso*-α-acid adsorbed on the membrane was calculated using the calibration curve obtained using the reference *iso*-α-acid sample. Figure 4 shows the relationship between the amount of adsorbed *iso*-α-acid and the lipid concentration of the membrane. The adsorption amount increased with the lipid concentration and reached a maximum, about 0.95 μg/cm^2^ around 0.1 wt % lipid concentration. The adsorption amount decreased when the lipid concentration was further increased to 1 wt %, but subsequently remained constant beyond this concentration.

### 3.4. Reference Potential

**Figure 5.**
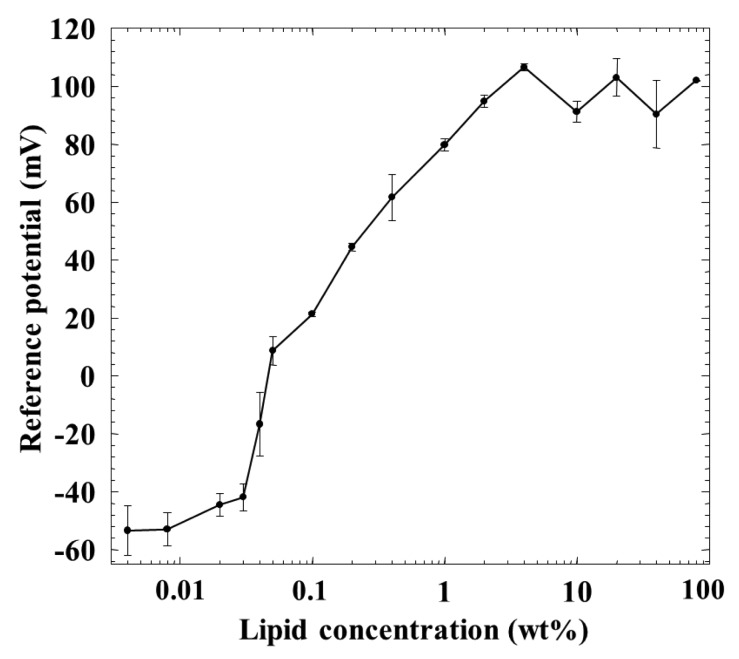
Relationship between the reference potential and the lipid concentration.

Figure 5 shows a relationship between the membrane potential for the reference solution 1 (reference potential) and the lipid concentration of the membrane. Although the reference potential increased with the lipid concentration, it became constant over 1 wt % lipid concentration. It showed negative values of about −50 mV at the lower lipid concentrations, and tended to show positive values beyond about 0.04–0.05 wt %, and increased to about 100 mV with the lipid concentration.

## 4. Discussion

The reason why the peak of the amount of *iso*-α-acid adsorbed onto the membrane appeared at the moderate lipid concentration of the membrane, shown in Figure 4, can be explained as follows, as also discussed in previous papers [2,21,22]: the amount of adsorption is affected by two factors: surface charge density and hydrophobicity of the membrane. As the concentration of positively charged lipid (TOMA) increases, the surface and interior charge densities of the lipid/polymer membrane increase but the hydrophobicity of the membrane decreases. *iso*-α-Acid is negatively charged in solution. When the membrane surface charge density increases, *iso*-α-acid tends to be attracted to the membrane surface by electrostatic interaction, resulting in an increased amount of *iso*-α-acid adsorbed onto the membrane. In contrast, there is another factor: the amount of *iso*-α-acid adsorbed onto the membrane decreases when the hydrophobicity of the membrane decreases. *iso*-α-Acid cannot be adsorbed onto the membrane surface or into the membrane interior in this situation. These opposite factors indicate that a peak should exist in the lipid concentration dependending of the amount of adsorbed *iso*-α-acid. In other words, the amount of adsorbed *iso*-α-acid increases because of the increased electrostatic attractive interaction and the moderate hydrophobicity of membrane in the region from low to moderate lipid concentration of the membrane. On the contrary, the adsorption amount decreases because of much decreased hydrophobicity in spite of the increased electrostatic interaction in the high-lipid-concentration region.

The reference potential increased with the lipid concentration, and then it became constant over 1 wt % lipid concentration, as shown in Figure 5. This reason can be considered as follows: generally speaking, the charge density affects the membrane potential in a logarithmic way [25–29]. When the charge density of the membrane increases with increasing lipid concentration, the membrane potential approaches the ideal Nernst potential [27,28]. In this situation, neither ionic flow nor salt flow tends to occur, and the membrane potential for the reference solution 1, *i.e.*, reference potential which is dominated by the surface potential cannot be affected by the charge density. Of course, lipid molecules TOMA, quaternary-ammonium-type lipid, dissociate fully at any pH but tend to be screened by counterions and hence apparently do not dissociate anymore due to the electrical repulsion when the lipid concentration becomes high, as is well known [25,26]. The reference potential should be negative in the low-lipid-concentration region, because the used plasticizer is negatively charged due to contained impurity, phenylphosphonic acid monooctyl ester [30]. Increasing positively charged lipids makes the surface and interior charge densities positive and causes the positive reference potential. It approaches the constant level of Nernst-type in the region of high charge density which appears at high lipid concentrations of the membrane.

This consideration can also be supported by the response of the membrane to saltiness, *i.e.*, the relative value for saltiness in Figure 2. The relative value was positive below 0.04 wt % lipid concentration and took zero around 0.04 wt %, and then turned to the negative value beyond this wt %; it took almost the constant value over 1 wt %. The relative value for saltiness depends on the charge density of the membrane [25–29]. Therefore, the dependences of the relative value for saltiness (Figure 2) and the reference potential (Figure 5) on the lipid concentration are quite similar with each other. The positively charged membrane responds to anions to the negative direction, and hence the sign of relative value is opposite to that of reference potential, as understood from Figures 2 and 5.

The dependence of the amount of adsorbed *iso*-α-acid on the lipid concentration in Figure 4 is somewhat similar to that of the CPA value for *iso*-α-acid in Figure 3, as characterized by a peak around 0.1 wt % lipid concentration. In the concentration region below 0.1 wt %, the adsorption amount and the CPA values behaved in the same way; *i.e.*, the CPA value reflects perfectly the amount of adsorption.

However, the amount of adsorption stayed constant while the CPA value (and also relative value) for *iso*-α-acid decreased at the lipid concentration higher than 1 wt %; *i.e.*, the monotonic decrease in CPA and relative values occurred in the lipid concentration region from 1 to 10 wt %. This region can be considered as the transient region of these response values. The amount of adsorbed *iso*-α-acid in Figure 4 and the reference potential in Figure 5 remained constant in this region. This seems contradictory. This apparent contradiction can be resolved as follows: the membrane potential change approaching the Nernst-type is not sensitive to the surface charge density in the higher-lipid-concentration region, as above. This consideration is supported by the response to saltiness of about −52 mV at 80 wt % lipid concentration (Figure 2), whose concentration is ten times that of reference solution; this response value is close to the ideal Nernst potential (−58 mV at 20 °C). Therefore, the increasing lipid concentration gradually increases the charge density, but this change is not reflected by the change in the membrane potential. When the same amount of *iso*-α-acid is adsorbed onto the membrane, the effects on the membrane potential, *i.e.*, the CPA and relative values, become larger for the membrane with lower charge density. As a result, a larger CPA and relative values were obtained at, e.g., 2 wt % concentration than at 10 wt % concentration. This result indicates that the CPA and relative values are affected by both the adsorption amount and the surface charge density.

In the wide region from 1 to 80 wt % lipid concentration of the membrane, the reference potential and the amount of *iso*-α-acid adsorbed onto the membrane kept constant. The reason why the adsorption amount does not change in this region can be considered to have no strong connection with the constant reference potential. In this region, the surface charge density increases slightly due to suppression of electrical repulsion and the hydrophobicity decreases slightly. It means that *iso*-α-acid tends to be attracted to the membrane a little more but does not become adsorbed anymore with increasing lipid concentration. It might be natural to consider that these opposite tendencies balance with each other.

This constant adsorption amount was already predicted by the previous papers [6,10,24], where the *iso*-α-acid concentration in solution which causes 10 mV membrane potential change was measured as a function of lipid concentration of the membrane. The linear relationship between the above *iso*-α-acid concentration and the lipid concentration was obtained. It implies that the amount of adsorption is proportional to the *iso*-α-acid concentration, irrespective of the lipid concentration, which agrees with the present result.

Last, whether *iso*-α-acid is adsorbed into the membrane interior or not can be explained as follows. The amount of adsorption is roughly 1 μg/cm^2^ for the membrane with 0.1 wt % lipid. In this case, the density of adsorbed *iso*-α-acid is estimated to be 1.66 × 10^15^/cm^2^. The *iso*-α-acid projection area is roughly 100 Å^2^, and hence the maximum number of *iso*-α-acid molecules which can be adsorbed per square centimeter is 10^14^. From these two estimates, therefore, we can consider that at least 16.6 layers, or roughly a few tens of layers, of *iso*-α-acid molecules are deposited on the surface and interior of the membrane for the 0.1 wt % case. The distribution coefficient (log*D*) of *iso*-α-acid is around 0.4 at weak acidic and neutral pH values. Therefore, it is very natural that *iso*-α-acid is adsorbed into the hydrophobic part of the membrane.

The *iso*-α-acid used is extracted from hops, and is considered to be a mixture of mainly *cis*- and *trans*-isohumulone. Alcohol drinks, represented by beer, contain several kinds of bitter substances like the above. Generally speaking, the taste sensor responds to these bitter and also other taste substances simultaneously at different levels or magnitudes. In some cases, the correlation between the sensor output and sensory evaluations is examined, and relatively good results have been obtained so far [1,2,4,6].

Other bitter substances, which are hydrophobic and negatively charged, can also be detected by the membrane studied here. Positively charged hydrophobic bitter or tasteless substances cannot affect the response of the membrane with high lipid (TOMA) concentrations because of electric repulsion. However, there is a possibility of signal interferences in CPA values if other negatively charged hydrophobic substances, which are not bitter, are present in a multi-hydrophobic component complex sample. At present, the detailed experiment has not been made, and hence it is left as a future task.

The temperature and the membrane structure such as porosity can affect the adsorption of *iso*-α-acid onto the membrane. Especially clarification of the relationship among the membrane structure, the relative value, the CPA value and the adsorption is very important for further improvement of taste sensor. At present, we are pursuing this subject by measurements of contact angle and chemical analyses using FTIR-RAS (Reflection Absorption Spectroscopy) and XPS (ESCA). The result will be reported in the near future. Whereas the response to *iso*-α-acid was examined in the present study, beer has other bitter substances such as β-acids (e.g., lupulone, colupulone, adlupulone). Research on the taste of these β-acids using a taste sensor is the next subject to be studied.

## 5. Conclusions

This study aimed at clarifying the relationship between the CPA value due to an acidic bitter substance, *iso*-α-acid, and the amount of *iso*-α-acid adsorbed onto lipid/polymer membranes used in a taste sensor. The amount of adsorption, which was obtained by spectroscopy, showed a maximum at the lipid (TOMA) concentration 0.1 wt %, and the same phenomenon was observed for the CPA value. The reason why the peaks appeared in both the CPA value and adsorption amount is due to the contradictory adsorption properties of *iso*-α-acid, which is attracted to the membrane via electrostatic interaction, but can hardly be adsorbed onto the membrane due to the decrease in the hydrophobicity with increasing lipid concentration. The amount of adsorption and the reference potential remained constant, while the CPA value decreased monotonically to zero in the 1–80 wt % concentration region. The constant amount of adsorption may be the result of a balance between increasing attractive electrostatic interactions and decreasing hydrophobic interactions. The decrease in CPA value despite the constant amount of adsorption is caused by decrease in the sensitivity of the membrane as the surface charge density increases. The amount of adsorption is affected by two factors—the surface charge density and the hydrophobicity of the membrane. The CPA value is also affected by two factors—the surface charge density and the amount of adsorption. Estimates of the amount of adsorption suggest that *iso*-α-acid molecules are adsorbed onto both the surface and interior of the membrane.

## Figures and Tables

**Table 1.** The composition of taste samples. Bitterness, umami and sweetness samples were obtained by adding a sample to reference solution as a solvent.

**Taste**	**Composition**
Bitterness	0.01 vol % *iso*-α-acid
Saltiness	300 mM KCl, 0.3 mM tartaric acid
Sourness	3 mM tartaric acid, 30 mM KCl
Umami	10 mM monosodium glutamate
Sweetness	300 mM sucrose
Reference solution	30 mM KCl, 0.3 mM tartaric acid
